# Efficacy and safety of acupoint autohemotherapy in treating stable chronic obstructive pulmonary disease: Protocol for a systematic review and meta-analysis

**DOI:** 10.1097/MD.0000000000017291

**Published:** 2019-09-20

**Authors:** Haidu Hong, Chushuan Huang, Chumin Chen, Rui Zhou, Jieying Li, Jianbo Liu, Xiaohong Liu

**Affiliations:** a The First Clinical College, Guangzhou University of Chinese Medicine; b The First Affiliated Hospital of Guangzhou University of Chinese Medicine, Guangzhou; c Clinical Medical College of Acupuncture Moxibustion and Rehabilitation, Guangzhou University of Chinese Medicine, China.

**Keywords:** acupoint autohemotherapy, chronic obstructive pulmonary disease, meta-analysis, protocol, systematic review

## Abstract

**Background:**

Chronic Obstructive Pulmonary Disease (COPD) is a clinically common chronic disease with the characteristic of recurrent attacks, difficulty of cure and high morbidity, disability, death rates. COPD exerts a great burden on patients, families and society. Acupoint Autohemotherapy (AA) is a traditional Chinese medicine (TCM) treatment by taking the patient's own venous blood and injecting them at acupoints, combined with the continuous stimulation of blood and the specific efficacy of the acupoint itself. It has been proved to be useful in pulmonary treatment and rehabilitation of COPD patients. However, the efficacy of AA on COPD patients has not been fully statistically evaluated. In this study, we aim to systematically examine the efficacy and safety of AA for COPD patients.

**Methods:**

Data from all English and Chinese databases, including Medline, Cochrane Library, Embase, China National Knowledge Infrastructure Database, Wanfang Database, China Biomedical Literature Database and Chongqing VIP information, will be used to conduct a systematic and comprehensive literature search. The range of date is from inception to July 2019. Randomized controlled trials (RCTs) related to AA and western medicine in the treatment of COPD will be included. Quality of included trials will be assessed according to the risk of bias tool of Cochrane Handbook 5.1.0. The GRADE approach will be used to rate the certainty of the evidence of estimates derived from meta-analysis. RevMan 5.3 will be used for data synthesis, sensitivity analysis, meta-regression analysis, subgroup analysis and risk of bias assessment. A funnel plot will be developed to evaluate reporting bias, and Begg and Egger tests will be used to assess funnel plot symmetries. Grading of recommendations assessment, development and evaluation system will be utilized to assess the quality of evidence.

**Results:**

This systematic review and meta-analysis aims to summarize the direct and indirect outcomes for AA and western medicine on COPD patients and evaluate its efficacy and safety. The results will be submitted to a peer-reviewed journal once completed.

**Conclusion:**

The systematic review will provide evidence to assess the efficacy and safety of AA and western medicine in the treatment of COPD patients.

**PROSPERO registration number:**

PROSPERO CRD42019137189

## 1 Introduction

Chronic Obstructive Pulmonary Disease (COPD) is a disease characterized by persistent respiratory symptoms and restricted airflow,^[1]^ and is a clinically common chronic disease with the characteristic of recurrent attacks, difficulty of cure and high morbidity, disability, death rates. It exerts a great burden on patients, families and society. According to reports, COPD is currently the fourth leading cause of death in the world.^[2]^ With the decline in environmental quality, it is expected to rise to the third place by 2020,^[3]^ becoming a health problem that cannot be ignored. However, the overall pathogenesis of COPD is not entirely clear, and the current treatments are mainly to relieve symptoms, which have significant limitations. Traditional Chinese medicine (TCM) has the advantages of multiple targets and systematic regulation, and its importance in the treatment of COPD has been highlighted.^[4]^

Autohemotherapy originates from Soviet in the 1940s and 1950s to treat tuberculosis hemoptysis by intramuscular injection, then it spread to China. Rui Jin, chief professor of Guangzhou University of TCM, created the Meridian Blood Injection Therapy which is also called Acupoint Autohemotherapy (AA) later, a TCM characteristic therapy that combine the continuous stimulation of blood with the action of acupoints to treat diseases by injecting the patient's venous blood into acupoints.^[5,6]^ It is a combination of TCM meridian theory, acupuncture, multi-component stimulation of blood cells and regulation of immune function of the body. Initially, the method was used to treat patients with malaise, malnutrition and anemia after malaria. After repeated clinical exploration, research and development, AA was used for more diseases treatments especially for respiratory diseases including COPD,^[7]^ asthma,^[8]^ cough variant asthma^[9]^ and has achieved satisfactory results.

At present, there are many clinical trials showing the efficacy and safety of AA combined with western medicine in the treatment of COPD. But the sample size varies, and it lacks certain persuasive power. The aim of this study is to comprehensively collect randomized controlled trials (RCTs) for the treatment of COPD with AA and western medicine, and to provide evidence for clinical practice by evaluating the efficacy and safety through the Cochrane systematic evaluation.

## 2 Methods

### 2.1 Study registering and reporting

The study protocol has been registered on PROSPERO (International Prospective Register of Systematic Reviews) (CRD42019137189). This protocol is developed in accordance with the Preferred Reporting Items for Systematic Reviews and Meta-analyses Protocols (PRISMA-P). Any protocol modifications made during the performing of the review will be recorded in the publication of the final report. The PRISMA Extension Statement is used to ensure all aspects of methods and findings are reported.

### 2.2 Eligibility criteria

The PICOS (Population-Intervention-Comparators-Outcomes-Study design) framework was adopted as the eligibility criteria for the review as follows.

#### 2.2.1 Study design

Whether or not blinding, allocation concealment, reporting withdrawal and loss of follow-up were performed, research that may be included in this review needs to first meet the following criteria:

1. Randomized controlled trial;
2. COPD patients without restrictions in sex, age, and race, and the ratio of forced expiratory volume in one second (FEV1) to forced vital capacity (FVC) was less than 70% or FEV1 was less than 80% of predicted values according to the 2019 Global Initiative for Chronic Obstructive Lung Disease (GOLD) criteria;
3. AA intervention lasted no less than 4 weeks;
4. Outcome measures included FEV1, FEV1%, FEV1/FVC%, AE, 6MWD, SGRQ, CRQ, mMRC, CAT, or BMI.

The following study designs or publication types will be excluded:

1. Patient diagnosed with COPD but has other diseases such as respiratory failure, bronchiectasis, bronchial asthma, lung cancer, etc.
2. Other interventions such as moxibustion, acupressure, Chinese medicine, etc. were combined in the treatment.
3. Duplicate or non-clinical research literature.
4. Containing other confusing signs or the data cannot be extracted, or lacking of outcome measures.

These trails can be adopted if multiple intervention data are available. If data for comparing multiple interventions is not directly available, we will attempt to obtain the raw data by sending an email to corresponding author of the article. If the data is not available at the end, these documents will also be excluded.

#### 2.2.2 Population

RCTs with a clear diagnosis of stable COPD were considered in accordance with the GOLD diagnostic criteria.^[10]^ The stable phase of COPD usually means that there is no acute exacerbation within 4 weeks. Patients were included regardless of nationality, gender, age, and ethnicity.

#### 2.2.3 Interventions

The control group was treated with western medicine. The main treatment option was using inhaled corticosteroids combined with bronchodilators, such as Salmeterol Xinafoateand Fluticasone Propionate Powder for inhalation and Budesonide and formoterol Fumarate Powder for Inhalation. At the same time, according to the patient's condition, using conventional therapy including bronchodilators, expectorants and other drugs combined with oxygen therapy, nutritional support therapy. The experimental group was treated with AA on the basis of the control group.

#### 2.2.4 Outcome measures

Primary outcome indicator: total effective rate of clinical treatment.

Secondary outcome indicator:

1. Pulmonary function:FEV1, FEV1%, FEV1/FVC%;
2. The incidence of Acute Exacerbations (AE);
3. Exercise endurance: 6-minutes walking distance(6MWD);
4. Health-related quality: St. George's respiratory questionnaire(SGRQ)and Chronic respiratory questionnaire(CRQ);
5. Health status: modified British medical research council (mMRC), COPD assessment test (CAT)and body mass index (BMI).

### 2.3 Data sources and search strategy

The work of document retrieval will be carried out from 3 English databases (Medline, Cochrane Library, Embase) and 4 Chinese databases (China National Knowledge Infrastructure Database, Wanfang Database, SinoMed, and Chongqing VIP information)from inception to July 2019. We will also undertake a targeted gray literature search on Clinical Trials.gov and the International Clinical Trials Registry Platform Search Portal to identify clinical trials, which are in progress or completed. And the following sources will also be searched including of Google Scholar, Web of Science and Baidu Scholar to identify trial protocols and other information. Furthermore, more studies will be identified after examining the reference lists of all retrieved articles.

These following combined texts and Medical Subject Headings (MeSH) terms are used by us to search in the sources: ’autologous blood’, ’autohemotherapy’, 'self-blood’, ’acupoint’, ’injection’, ’chronic obstructive pulmonary disease’, ’COPD’, ’pulmonary disease’. The search terms will be ’autologous blood’ OR ’autohemotherapy’ OR 'self-blood’ AND ’acupoint’ OR ’injection’ AND ’chronic obstructive pulmonary disease’ OR ’COPD’ OR ’pulmonary disease’.

### 2.4 Study selection and data extraction

**Figure 1:**
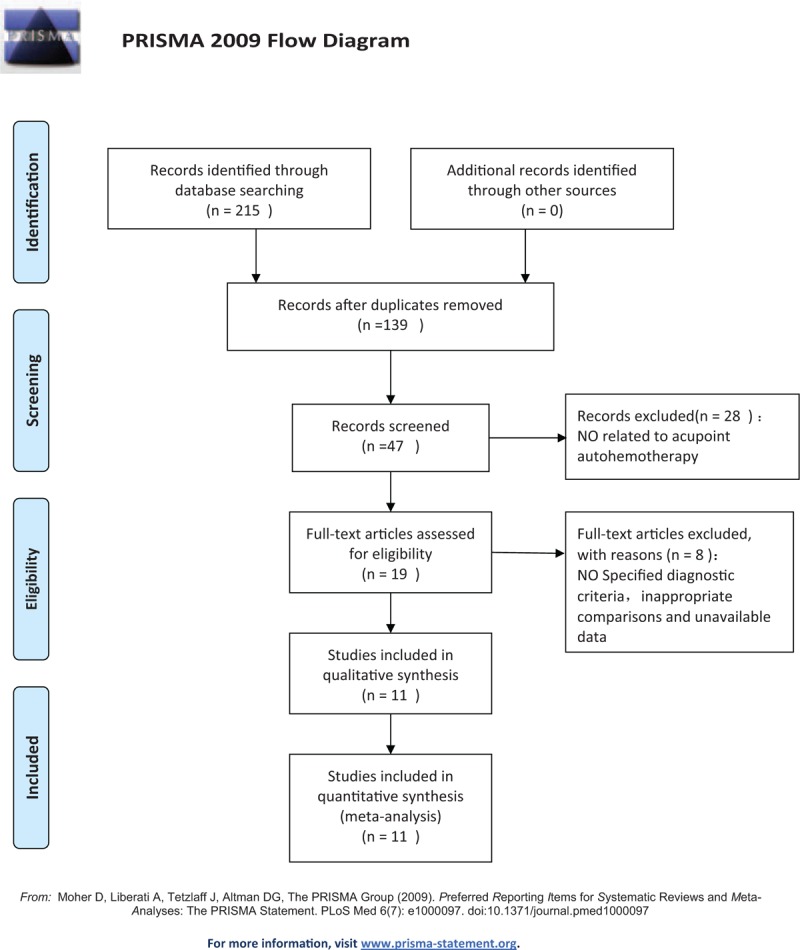
Flow diagram of study selection process.

Two researchers (Chushuan Huang, Rui Zhou) extracted data independently from the literature downloaded from 7 databases and managed by EndNote X9. Two researchers (Chumin Chen, Rui Zhou) independently screened the included studies, extracted data, evaluated the quality of included studies and cross-checked with each other according to the established selection criteria. If there are disagreements about the articles between the 2 researchers, objections will be delivered to the third researcher (Haidu Hong) and final decision will be made by him. First, the preliminary screening will be performed by reading the title and abstract of the obtained literature. Studies that fail to meet the eligibility criteria will be excluded. Then, full text of the articles will be retrieved to further determine whether they are included. The screening process will be presented with reference to the PRISMA statement as Figure 1. Microsoft Excel will be used to extract data and collect relevant information. A self-made data extraction form will be used to extract data, including the first author's name, year of publication, study design, intervention, and control group information, sample size, duration of intervention, and outcomes, including FEV1, FEV1%, FEV1/FVC%, AE, 6MWD, SGRQ, CRQ, mMRC, CAT, and BMI results. We contacted the corresponding authors for additional information if necessary.

### 2.5 Quality assessment

The Risk of Bias Tool (ROB) in Cochrane Handbook (5.1.0) will be used to assess the methodological quality of included trials by two independent reviewers (Chumin Chen, Rui Zhou). Disagreements will be resolved by discussing with a third reviewer (Xiaohong Liu). The ROB contains the following seven items: random sequence generation, allocation concealment, blinding of participants and personnel, blinding of outcome assessment, incomplete outcome data, selective reporting and other sources of bias. The judgment of each item is divided into three levels: low risk of bias, high risk of bias and unclear risk of bias.

We will evaluate the quality of evidence of the included studies through the Grading of Recommendations Assessment, Development and Evaluation (GRADE) system. The limitations of the study, inconsistencies, indirect evidence, inaccuracies and publication bias will also be considered. Four levels of quality of evidence will be used: high, moderate, low, or very low.

### 2.6 Dealing with missing data

If necessary, we will contact both senior and/or corresponding author of articles through email or telephone for further information about any missing data or unclear measurement scales. If no one responds or sufficient information cannot be obtained in this way, we will analyze the available data. The potential impact of insufficient data on the review results will be took into account in the discussion section.

### 2.7 Statistical analysis

The statistical analysis will be performed according to their commendations of the cochrane handbook and using the software of cochrane collaboration, RevMan 5. 3, available from the cochrane website. All outcomes will be continuous variables. The standardized mean difference and its 95% CIs will be calculated. Initially, a fixed-effect model will be used to compare with the outcomes expressed in the same scale. The heterogeneity of the effects of trials will be evaluated by the Q^2^ test and the I^2^ test. Heterogeneity will be considered as substantial if the I^2^ statistic≥50% and *P* < .10. If heterogeneity is considered as substantial, reasons for this heterogeneity will be searched for and a random-effect model could be used for comparison.

Sensitivity analysis, meta-regression, and subgroup analysis might be conducted if there are potential sources of heterogeneity. Qualitative synthesis will be performed if data extraction is insufficient.

### 2.8 Patient and public involvement

This part is not covered in this study.

## 3 Ethics and dissemination

We aim to publish this systematic review in a peer-reviewed journal. This study aims at providing available evidence for the efficacy and safety of AA on the treatment of stable COPD patients. Ethical approval will not be demanded since there is no participant privacy involved.

## 4 Discussion

The latest research shows that the overall prevalence of COPD in China is 8.6%, while the subgroup of patients over 40-year-old has nearly 99 million people, accounting for 13.7%.^[11]^ If the COPD is not controlled, it will develop into the final outcome of chronic pulmonary heart disease. At present, the main treatment methods of Western medicine included smoking cessation, oxygen inhalation, lung volume reduction surgery, and drug treatment (inhalation, oral administration, and systemic administration). However, there are still controversies and limitations in the development of individualized programs, the use of bronchodilators in the initial treatment, the side effects and withdrawal of ICS and so on.^[12]^

COPD belongs to the category of ’lung distention’ in TCM theory, which is due to repeated episodes of chronic lung diseases, prolonged unhealed, resulting in lung, spleen, kidney 3 visceral damage, lung qi stagnation, chest fullness.^[13]^ The theory of AA comes from the TCM mainly. First, blood can nourish the weak state of the body, and the meridians can connect with the major organs of the body and be effective through specific acupoints. Second, the lungs lead the blood vessels and veins, so treatment with blood can be effectively applied in lung diseases. Third, the stimulation produced by acupuncture and blood synergy is long-lasting, slow, continuous and effective.^[14]^ Modern research shows that the human body's own blood is rich in hormones, antibodies, complement and other elements, does not have rejection and can stimulate the body to release more immunoglobulin, enhance microcirculation. At the same time, it antagonizes histamine, acetylcholine and serotonin to reduce the permeability of capillaries and inhibit allergic reactions.^[15,16]^ Therefore, based on the theoretical basis of Chinese and Western medicine, AA is particularly suitable for improving the long-term weakness of COPD. In terms of treatment, the back-three-needles (BL13, BL11, BL12), EX-B1 and other pulmonary meridian acupoints are mainly used.^[17]^ According to the different symptoms of the patients, other auxiliary acupoints such as BL20 and ST36 are appropriately included.^[18]^

Although a large number of studies have shown that AA is effective in treating patients with COPD, the evidence for AA in improving FEV1, 6MWD and reducing the number of acute attacks is still insufficient. In order to systematically assess the effect of AA on all aspects of COPD treatment, our goal is to include adequate research on meta-analysis to ensure sufficient evidence. We expect AA to have a more positive impact on COPD patients. The results of this review may help provide more reliable evidence for the advancement of AA management and application in the treatment of COPD.

## Author contributions

**Conceptualization:** Haidu Hong, Chushuan Huang, Jieying Li.

**Data curation:** Chushuan Huang, Chumin Chen, Rui Zhou.

**Formal analysis:** Chumin Chen, Rui Zhou, Jieying Li.

**Funding acquisition:** Rui Zhou, Xiaohong Liu.

**Investigation:** Haidu Hong, Chushuan Huang, Jieying Li.

**Methodology:** Chushuan Huang.

**Project administration:** Chumin Chen.

**Resources:** Chumin Chen, Rui Zhou.

**Software:** Haidu Hong, Chushuan Huang.

**Supervision:** Rui Zhou, Xiaohong Liu.

**Visualization:** Chushuan Huang.

**Writing – original draft:** Haidu Hong, Chushuan Huang, Xiaohong Liu.

**Writing – review & editing:** Haidu Hong.
